# Genetic diversity analysis and DNA fingerprint construction of *Zanthoxylum* species based on SSR and iPBS markers

**DOI:** 10.1186/s12870-024-05373-1

**Published:** 2024-09-07

**Authors:** Xiaoxi Zhang, Wei Chen, Zhiwu Yang, Chengrong Luo, Weiwei Zhang, Feng Xu, Jiabao Ye, Yongling Liao

**Affiliations:** 1 College of Horticulture and Gardening, Yangtze University, Jingzhou, Hubei 434025 China; 2 Sichuan Academy of Forestry, Chengdu, Sichuan 610081 China

**Keywords:** *Zanthoxylum*, SSR markers, iPBS markers, Genetic diversity, Genetic structure, DNA fingerprint

## Abstract

*Zanthoxylum* is a versatile economic tree species utilized for its spice, seasoning, oil, medicinal, and industrial raw material applications, and it has a lengthy history of cultivation and domestication in China. This has led to the development of numerous cultivars. However, the phenomenon of mixed cultivars and confusing names has significantly obstructed the effective utilization of *Zanthoxylum* resources and industrial development. Consequently, conducting genetic diversity studies and cultivar identification on *Zanthoxylum* are crucial. This research analyzed the genetic traits of 80 *Zanthoxylum* cultivars using simple sequence repeat (SSR) and inter-Primer Binding Site (iPBS) molecular markers, leading to the creation of a DNA fingerprint. This study identified 206 and 127 alleles with 32 SSR markers and 10 iPBS markers, respectively, yielding an average of 6.4 and 12.7 alleles (*Na*) per marker. The average polymorphism information content (*PIC*) for the SSR and iPBS markers was 0.710 and 0.281, respectively. The genetic similarity coefficients for the 80 *Zanthoxylum* accessions ranged from 0.0947 to 0.9868 and from 0.2206 to 1.0000, with mean values of 0.3864 and 0.5215, respectively, indicating substantial genetic diversity. Cluster analysis, corroborated by principal coordinate analysis (PCoA), categorized these accessions into three primary groups. Analysis of the genetic differentiation among the three *Zanthoxylum* (*Z. bungeanum*, *Z. armatum*, and *Z. piperitum*) populations using SSR markers revealed a mean genetic differentiation coefficient (*Fst*) of 0.335 and a gene flow (*Nm*) of 0.629, suggesting significant genetic divergence among the populations. Molecular variance analysis (AMOVA) indicated that 65% of the genetic variation occurred within individuals, while 35% occurred among populations. Bayesian model-based analysis of population genetic structure divided all materials into two groups. The combined *PI* and *PIsibs* value of the 32 SSR markers were 4.265 × 10^− 27^ and 1.282 × 10^− 11^, respectively, showing strong fingerprinting power. DNA fingerprints of the 80 cultivars were established using eight pairs of SSR primers, each assigned a unique numerical code. In summary, while both markers were effective at assessing the genetic diversity and relationships of *Zanthoxylum* species, SSR markers demonstrated superior polymorphism and cultivar discrimination compared to iPBS markers. These findings offer a scientific foundation for the conservation and sustainable use of *Zanthoxylum* species.

**Supplementary Information:**

The online version contains supplementary material available at 10.1186/s12870-024-05373-1.

## Introduction

*Zanthoxylum* L., a member of the Rutaceae family, is a small evergreen or deciduous tree, shrub, or woody vine. There are approximately 250 species worldwide, primarily found in the tropical and subtropical regions of East Asia and North America [1]. Specifically, China is home to 45 species, 13 varieties, and 2 formas distributed in both the northern and southern regions. The predominant cultivated species in China are *Zanthoxylum bungeanum* Maxim. and *Zanthoxylum armatum* DC., commonly referred to as “Huajiao” or “Chinese pepper,” which are used as edible spices [2–4]. Moreover, *Zanthoxylum* species have a wide range of applications, including in food, medicine, ornamental purposes, and soil and water conservation, demonstrating significant economic and ecological benefits.

China serves as the leading producer of *Zanthoxylum*, boasting the highest yield and cultivation area globally. Furthermore, China has been at the forefront of utilizing and domesticating *Zanthoxylum* species, with records indicating its use dating back to the 11th to 10th centuries BC [5]. Over the course of extensive cultivation and domestication, a diverse range of *Zanthoxylum* cultivars and types have emerged. As the cultivation area expands and the exchange of resources between different *Zanthoxylum* production regions becomes more frequent, the genetic background of *Zanthoxylum* has become increasingly complex. Additionally, varying classification criteria in different regions have contributed to issues such as cultivar confusion and name ambiguity. Consequently, instances of synonymy, homonymy, and substandard materials often arise in the cultivation and commercial circulation of *Zanthoxylum*. Morphological identification methods based solely on phenotypic traits prove inadequate for distinguishing these similar materials. This not only compromises the rights and interests of consumers, growers, and breeders but also hinders the development and utilization of *Zanthoxylum* germplasm resources and the process of cultivar selection [3, 6]. Therefore, conducting extensive research on genetic diversity analysis, genetic map construction, and cultivar identification techniques for *Zanthoxylum* is highly important. This research will play a crucial role in safeguarding the development of *Zanthoxylum* germplasm resources and ensuring the healthy growth of the industry.

Molecular markers are extensively utilized in genetic diversity analysis, germplasm resources identification, and genetic map construction of species. Among the various molecular marker technologies available, SSR has gained wide popularity due to their high polymorphism level, reliable repeatability, codominance, and multiple allele variations. It has been chosen as the preferred method for constructing plant DNA fingerprints by the International Union for the Protection of New Plant Varieties (UPOV) [7, 8]. In recent years, several molecular markers have been applied in the study of *Zanthoxylum*. Li et al. [9] conducted the first genome-wide survey of *Zanthoxylum* and used 36 Genomic-SSR (G-SSR) markers, which demonstrated polymorphism, to classify 15 *Zanthoxylum* cultivars into two categories. Using three candidate DNA barcode regions (ITS2, ETS, and trnH psbA), Zhao et al. [10] identified 69 materials representing 13 Chinese pepper species. Feng et al. on the other hand, employed SRAP [3], chloroplast DNA (cpDNA) [4], EST-SSR [11], ISSR [12], and SNP [13] markers to analyze the genetic diversity, phylogenetic relationships, and genetic structure of *Zanthoxylum* species. Although numerous SSR markers have been identified in *Zanthoxylum* species, their potential for use in identifying *Zanthoxylum* germplasm resources has not been validated.

iPBS (inter-Primer Binding Site), proposed in 2010 by Kalendar et al. [14], is a novel molecular marker technology for polymorphism amplification based on reverse transcription transposon sequences. Compared to other molecular marker techniques, iPBS does not require sequence information or primer design in advance. The detection of produced markers can be achieved through agarose gel electrophoresis, a simple, fast, and cost-effective method. The primers used in iPBS are versatile and can be utilized in a wide range of plants and animals. Moreover, iPBS exhibits high polymorphism and reproducibility [14, 15]. As a result of these advantages, iPBS has been increasingly employed in plants for evaluating genetic diversity, as observed in grape [16], safflower (*Carthamus tinctorius*) [17], and bamboo [18] studies. However, so far, there are no reports on the application of iPBS as a molecular marker in *Zanthoxylum*. Notably, a study by Hu et al. [19] revealed that approximately 71.2% of the *Z. armatum* genome and 70.6% of the *Z. bungeanum* genome consisted of LTR-type reverse transcriptional transposons. Consequently, the reverse transcriptional transposon-based marker approach seems appealing as a tool for fingerprinting *Zanthoxylum* species.

In this study, we assessed the genetic diversity of 80 *Zanthoxylum* accessions using both SSR and iPBS molecular markers. Through this analysis, we constructed DNA fingerprints to provide a reference for the assessment of resources and cultivar identification of *Zanthoxylum*. Furthermore, this research endeavors to establish a scientific foundation for the utilization of *Zanthoxylum* resources and the protection of intellectual property rights.

## Materials and methods

### Plant materials and DNA extraction

Eighty plant samples including three *Zanthoxylum* species (*Z. bungeanum*, *Z. armatum*, *Z. piperitum*) were collected from the *Zanthoxylum* Germplasm Resource Bank in Hanyuan County, Sichuan Province (Table 1). The sampling process involved selecting well-growing *Zanthoxylum* species plants, randomly selecting 3 individual samples from each cultivar, and collecting fresh and pest-free *Zanthoxylum* leaves. These leaves were stored in a -80 °C freezer for future use.

**Table 1:** List of 80 *Zanthoxylum* accessions used in the present study

Code	Cultivar or common name	Abbreviation	Species	Provinance
1	Hanchengdangcunwuci	HCDCWC	*Z. bungeanum*	Shanxi
2	Hanchengxiaohongpao	HCXHP	*Z. bungeanum*	Shanxi
3	Hanchengyexuanyihao	HCYXYH	*Z. bungeanum*	Shanxi
4	Hanchengwuci	HCWC	*Z. bungeanum*	Shanxi
5	Hanchenghuajiao	HCHJ	*Z. bungeanum*	Shanxi
6	Hanchengwuciyihao	HCWCYH	*Z. bungeanum*	Shanxi
7	Fengxiandahongpao	FXDHP	*Z. bungeanum*	Shanxi
8	Gelaoxibeinongyehuajiao	GLXBNYHJ	*Z. bungeanum*	Shanxi
9	Fuguhuajiao	FGHJ	*Z. bungeanum*	Shanxi
10	Xingqinyihao	XQYH	*Z. bungeanum*	Shanxi
11	Xingqinerhao	XQEH	*Z. bungeanum*	Shanxi
12	Hanchengputaohuajiao	HCPTHJ	*Z. bungeanum*	Shanxi
13	Germany Huajiao	GHJ	*Z. bungeanum*	Germany
14	Guojiadahongpao	GJDHP	*Z. bungeanum*	Gansu
15	Qinanhuajiao	QAHJ	*Z. bungeanum*	Gansu
16	Xinongwuci	XNWC	*Z. bungeanum*	Gansu
17	Wududahongpao	WDDHP	*Z. bungeanum*	Gansu
18	Linxiamianjiao	LXMJ	*Z. bungeanum*	Gansu
19	Qinanyihao	QAYH	*Z. bungeanum*	Gansu
20	Longnanbayuejiao	LNBYJ	*Z. bungeanum*	Gansu
21	Nanqiangyihao	NQYH	*Z. bungeanum*	Gansu
22	Longnanqiyuejiao	LNQYJ	*Z. bungeanum*	Gansu
23	Bayuejiao	BYJ	*Z. bungeanum*	Gansu
24	Shizitou	SZT	*Z. bungeanum*	Gansu
25	Longnandahongpao	LNDHP	*Z. bungeanum*	Gansu
26	Baishajiao	BSJ	*Z. bungeanum*	Hebei
27	Doujiao	DJ	*Z. bungeanum*	Gansu
28	Xiheyoujiao	XHYJ	*Z. bungeanum*	Gansu
29	Hanyuanhuajiao	HYHJ	*Z. bungeanum*	Sichuan
30	Hanyuanwuci ♂	HYWCXZ ♂	*Z. bungeanum*	Sichuan
31	Hanyuanwuci ♀	HYWCCZ ♀	*Z. bungeanum*	Sichuan
32	Hanyuanzaoshu	HYZS	*Z. bungeanum*	Sichuan
33	Hanyuanwanshuyihao	HYWSYH	*Z. bungeanum*	Sichuan
34	Shujiaoerhao	SJEH	*Z. bungeanum*	Sichuan
35	Shujiaosanhao	SJSH	*Z. bungeanum*	Sichuan
36	Dahongpaowang	DHPW	*Z. bungeanum*	Sichuan
37	Mianyangwuciqinghuajiao	MYWCHJ	*Z. armatum*	Sichuan
38	Jinquanwuci	JQWC	*Z. bungeanum*	Sichuan
39	Yuexihuajiao	YXHJ	*Z. bungeanum*	Sichuan
40	Maoxianliuyuejiao	MXLYJ	*Z. bungeanum*	Sichuan
41	Maoxianqiyuejiao	MXQYJ	*Z. bungeanum*	Sichuan
42	Nanludahongpao	NLDHP	*Z. bungeanum*	Sichuan
43	Dahongpao	DHP	*Z. bungeanum*	Sichuan
44	Zanghongjiao	ZHJ	*Z. bungeanum*	Sichuan
45	Xizanghuajiao	XZHJ	*Z. bungeanum*	Xizang
46	Laiwuxiaohongpao	LWXHP	*Z. bungeanum*	Shandong
47	Laiwudahongpao	LWDHP	*Z. bungeanum*	Shandong
48	Jiningzouchenghuajiao	JNZCHJ	*Z. bungeanum*	Shandong
49	Hebeiwuci	HBWC	*Z. bungeanum*	Hebei
50	Hebeixinglonghuajiao	HBXLHJ	*Z. bungeanum*	Hebei
51	Hebeizhengluhuajiao	HBZLHJ	*Z. bungeanum*	Hebei
52	Linzhouhonghuajiao	LZHHJ	*Z. bungeanum*	Henan
53	Pingshundahongpao	PSDHP	*Z. bungeanum*	Shanxi
54	Ruichenghuajiao	RCHJ	*Z. bungeanum*	Shanxi
55	Zhenxiongxuejiao	ZXXJ	*Z. bungeanum*	Yunnan
56	Zhenxionghuajiao	ZXHJ	*Z. bungeanum*	Yunnan
57	Zhaotongdahongpao	ZTDHP	*Z. bungeanum*	Yunnan
58	Jinjiangyihao	JJYH	*Z. armatum*	Sichuan
59	Neijiangqinghuajiao	NJQHJ	*Z. armatum*	Sichuan
60	Meishanqinghuajiao	MSQHJ	*Z. armatum*	Sichuan
61	Hanyuanputaoqingjiao	HYPTQJ	*Z. armatum*	Sichuan
62	Pengxiqinghuajiao	PXQHJ	*Z. armatum*	Sichuan
63	Hongyatengjiao	HYTJ	*Z. armatum*	Sichuan
64	Jinyangqinghuajiao	JYQHJ	*Z. armatum*	Sichuan
65	Guanganqinghuajiao	GAQHJ	*Z. armatum*	Sichuan
66	Qingjinyihao	QJYH	*Z. armatum*	Sichuan
67	Yaojiao	YJ	*Z. armatum*	Sichuan
68	Cijiao	CJ	*Z. bungeanum*	Sichuan
69	Zhaotongzhuyejiao	ZTZYJ	*Z. armatum*	Yunnan
70	Wucitengjiao	WCTJ	*Z. armatum*	Chongqing
71	Jiuyeqinghuajiao	JYQHJ	*Z. armatum*	Chongqing
72	Huapinghuajiao	HPHJ	*Z. armatum*	Yunnan
73	Yongqingyihao	YQYH	*Z. armatum*	Yunnan
74	Luqingyihao	LQYH	*Z. armatum*	Yunnan
75	Putaoshanjiao	PTSJ	*Z. pipertum*	Japan
76	Zhaocangshanjiao	ZCSJ	*Z. pipertum*	Japan
77	Liujinshanjiao	LJSJ	*Z. pipertum*	Japan
78	Japan Wuciyihao	JWCYH	*Z. pipertum*	Japan
79	Huashanjiao	SHJ	*Z. pipertum*	Japan
80	Zhaocangshanjiao ♂	ZCSJ ♂	*Z. pipertum*	Japan

Following the method outlined by Porebski et al. [20], DNA was extracted using a modified CTAB method. The concentration and purity of the extracted DNA were subsequently assessed using a NanoDrop One Ultra-Micro UV Spectrophotometer (Thermo Fisher Scientific Inc., USA). The integrity of the DNA was verified through 1% agarose gel electrophoresis. The DNA was uniformly diluted to a concentration of 100 ng/µl and stored in a -40 °C refrigerator as a backup.

### SSR primer screening and PCR amplification

Six hundred pairs of primers were selected from the G-SSR primers developed in the previous stage of our group, containing dinucleotide, trinucleotide, tetranucleotide, pentanucleotide, hexanucleotide and complex types of SSR sites, and all of them were tested for specificity and synthesized by Sangon Biotech (Shanghai) Co., Ltd. These primers were used to amplify DNA from seven *Zanthoxylum* accessions (FGHJ, HYWCXZ ♂, SJSH, NLDHP, YJ, WCTJ, JYQHJ) (Table 1) that exhibited significant morphological differences. Primers with clear target bands, simple band types, and high polymorphism were selected.

PCR reaction system (25 µL): 3G Taq Master Mix for PAGE (Red Dye) (Nanjing Vazyme Biotech Co., Ltd.)12.5 µL; forward and reverse primers: 1.0 µL (10 pmol/L); DNA 100 ng; fill with ddH_2_O to 25.0 µL. PCR amplification was performed using Touchdown PCR method, with a reaction procedure of pre-denaturation at 95℃ for 6 min; denaturation at 95 °C for 15 s, annealing at 64 °C for 15 s (thereafter, cycling at 64 °C ∼ 54 °C for every 2 °C decrease until 54 °C), and extension at 72 °C for 30 s; denaturation at 95 °C for 15 s, annealing at 54 °C for 15 s, extension at 72 °C for 30 s, and cycling 25 times; extend at 72 °C for another 5 min and stored at 4 °C.

PCR products were detected by 10% nondenaturing polyacrylamide gel electrophoresis at 185 V for 130 min. After silver staining and color development, they were photographed with a camera.

### iPBS primer screening and PCR amplification

Eighty-three iPBS primers published by Kalendar et al. [14] were synthesized, and these primers were amplified by PCR using DNA from *Zanthoxylum* accessions (FGHJ, HYWCXZ ♂, SJSH, NLDHP, YJ, WCTJ, JYQHJ) (Table 1) that exhibited significant morphological differences, and those with clear amplified bands, high polymorphism, and high stability were selected. 83 iPBS primers were synthesized by Sangon Biotech (Shanghai) Co., Ltd.

PCR reaction system (25 µL): 2 × Rapid Taq Master Mix (Nanjing Vazyme Biotech Co., Ltd.) 12.5 µL, iPBS primer 1.0 µL (10 pmol/L), ddH_2_O 10.5 µL, DNA 1.0 µL. Reaction procedure: Pre-denaturation at 95 °C for 6 min; denaturation at 95 °C for 15 s, annealing at 39.0 ∼ 65.0 °C for 30 s, extension at 72 °C for 1 min, 32 cycles; complete extension at 72 °C for 5 min, stored at 4 °C.

PCR products were detected by 1.2% agarose gel electrophoresis at 100 V for 28 min, and photographed by a gel imaging system at the end of electrophoresis.

### Data statistics and analysis

The bands in the SSR and iPBS electrophoresis profiles were counted using Excel 2019 and assigned corresponding “1” or “0” values based on the presence or absence of bands, respectively. These data were used to create a two-dimensional matrix of “0, 1”.

For SSR markers, the data formats were converted using DataFormater software [21]. Genetic parameters such as number of observed alleles (*Na*), number of effective alleles (*Ne*), Shannon’s information index (*I*), expected heterozygosity (*He*), observed heterozygosity (*Ho*), fixed coefficient of population genetic differentiation (*Fst*), gene flow (*Nm*), probability of identity (*PI*), and probability of identity among siblings (*PIsibs*) were computed by GenAlex 6.503 software [22]; and the test materials were principal coordinate analysis (PCoA) and analysis of molecular variance (AMOVA) were performed. The polymorphism information content (*PIC*) of SSR primers was calculated using PIC-Calc 0.6 software. Genetic similarity coefficients (*GS*) among the test materials were calculated using NTSYS-pc 2.1 software [23], and the unweighted pair-group method with arithmetic means (UPGMA) in the SAHN module was used for cluster analysis and construction of dendrograms. Population structure analysis was performed by Structure 2.3.2 software [24] with the following parameters: Length of Burin Period = 50,000, Number of MCMC Reps after Burnin = 100,000, K = 1 ∼ 10, and 5 replications for each K value; the results were uploaded to the Structure Harvester website (https://taylor0.biology.ucla.edu/structureHarvester/) to determine the optimal K value; the results corresponding to the optimal K value were subsequently analyzed by repeated sampling through the CLUMPP program; and finally visualized using the distrut program.

For iPBS markers, observed alleles (*Na*), number of effective alleles (*Ne*), Shannon’ s information index (*I*), and Nei’ s gene diversity (*H*) were calculated for amplified loci and populations by PopGene 1.32 software [25]; PCoA and cluster analysis based on UPGMA method were performed using NTSYS-pc 2.1 software. Since the iPBS markers are dominant markers, the *PIC* was calculated with reference to the method of Hinze et al. [26]: *PIC*_i_ = 1 - (*p*^2^ + *q*^2^), where *p* is the frequency of “1” appearing in the i-th band of the primer and q is the frequency of “0” appearing in the i-th band of the primer; when *p* = *q* = 0.5, the *PIC* value of the dominant marker is the largest (0.5), and the polymorphism of the primer is the highest.

### Construction of DNA fingerprint

The SSR primers for constructing fingerprints were screened according to the following conditions: (1) The amplified bands are clear, and the results are stable and reproducible; (2) Primers with high *PIC* and low *PI* values; (3) The principle of identifying the most materials with the fewest number of primers was followed; (4) Ensure the uniqueness of the fingerprint of each accession.

The band information amplified by each primer was recorded in Excel 2019 using “0”, “1”, and “9” to signify “no band,” “with band,” and “no amplification,“, respectively, to form a digital fingerprint map. Subsequently, the information (name, Latin name, cultivar types, provenance) of each *Zanthoxylum* accession was integrated with its fingerprint code and imported into the “Caoliao QR Code” online software (https://cli.im/) to generate QR codes for the fingerprints of 80 *Zanthoxylum* cultivars.

## Results

### SSR primer screening and genetic diversity of the markers

A total of 32 pairs of polymorphic SSR primers (Supplementary Table S1) were screened from 600 pairs of primers using seven *Zanthoxylum* accessions with significant morphological differences. These primers were subsequently used to amplify all peppercorn samples.

A total of 206 (*Na*) of the 32 pairs of SSR primers were detected in 80 *Zanthoxylum* accessions. The average number of alleles detected per pair of primers ranged from 3.000 (D27, T6) to 11.000 (P.17), with an average value of 6.438 (Table 2). This finding suggested that the tested *Zanthoxylum* accessions exhibit relatively abundant allelic variation. The number of effective alleles (*Ne*) varied from 1.648 (P4.2) to 6.181 (D86), with a mean value of 3.254. Observed heterozygosity (*Ho*) and expected heterozygosity (*He*) values indicate the magnitude of genetic variance for different SSR primers, with higher *Ho* values indicating higher heterozygosity. Among the 32 markers, the *Ho* values ranged from 0.225 (D112) to 0.950 (D39), and the He values ranged from 0.393 (P4.2) to 0.838 (D86). The mean values for *Ho* and *He* were 0.638 and 0.661, respectively. Shannon’s information index (*I*) varied from 0.677 (P4.2) to 1.937 (D86), with a mean value of 1.336. These results indicate that the tested *Zanthoxylum* materials exhibit a high degree of genetic variation and rich genetic diversity.

**Table 2:** The genetic diversity statistics of 32 SSR markers in 80 *Zanthoxylum* accessions

Marker ID	Na	Ne	I	Ho	He	Nm	PIC	PI	PIsibs
D11	7.000	4.441	1.597	0.613	0.775	0.452	0.827	0.086	0.384
D23	10.000	4.385	1.749	0.821	0.772	0.346	0.706	0.079	0.384
D27	3.000	1.875	0.686	0.675	0.467	0.457	0.657	0.388	0.614
D39	6.000	3.742	1.496	0.950	0.733	1.299	0.735	0.108	0.411
D49	7.000	2.584	1.240	0.600	0.613	1.527	0.738	0.193	0.492
D50	6.000	3.053	1.329	0.797	0.672	0.801	0.710	0.150	0.451
D79	6.000	2.550	1.205	0.663	0.608	0.438	0.649	0.192	0.494
D81	6.000	4.452	1.611	0.900	0.775	3.964	0.793	0.084	0.383
D86	8.000	6.181	1.937	0.688	0.838	0.355	0.785	0.046	0.342
D93	5.000	3.579	1.385	0.465	0.721	0.206	0.813	0.127	0.421
D106	4.000	2.321	1.062	0.588	0.569	0.247	0.665	0.234	0.524
D111	4.000	2.463	1.027	0.600	0.594	0.665	0.621	0.249	0.515
D112	4.000	1.718	0.750	0.225	0.418	0.363	0.484	0.389	0.638
F31	6.000	3.923	1.520	0.888	0.745	0.290	0.777	0.104	0.404
F84	8.000	2.949	1.336	0.675	0.661	0.246	0.819	0.165	0.461
F86	5.000	2.629	1.139	0.800	0.620	0.441	0.722	0.214	0.494
T16	3.000	2.205	0.886	0.300	0.547	0.063	0.649	0.294	0.550
T78	6.000	3.188	1.375	0.688	0.686	0.300	0.709	0.142	0.442
T83	4.000	2.101	0.951	0.575	0.524	0.212	0.795	0.284	0.559
T86	10.000	3.740	1.660	0.658	0.733	0.213	0.710	0.100	0.409
N63	7.000	3.571	1.401	0.603	0.720	0.289	0.800	0.129	0.422
N76	5.000	2.732	1.194	0.861	0.634	0.631	0.656	0.184	0.479
P3.16	6.000	3.236	1.362	0.550	0.691	0.301	0.632	0.147	0.441
P4.2	5.000	1.648	0.677	0.413	0.393	1.442	0.400	0.428	0.660
P4.11	9.000	4.967	1.782	0.608	0.799	0.368	0.803	0.069	0.368
P4.17	11.000	4.110	1.774	0.632	0.757	0.398	0.746	0.084	0.393
P4.19	10.000	4.778	1.795	0.538	0.791	0.289	0.739	0.072	0.373
P5.10	6.000	3.421	1.399	0.658	0.708	0.239	0.572	0.135	0.430
P6.20	7.000	3.365	1.505	0.411	0.703	0.154	0.806	0.119	0.428
P6.27	7.000	2.553	1.294	0.810	0.608	1.796	0.703	0.186	0.492
P6.30	6.000	3.353	1.350	0.808	0.702	1.131	0.807	0.143	0.435
C6	9.000	2.325	1.279	0.367	0.570	0.206	0.676	0.209	0.517
Total	206.000	104.140	42.751	20.423	21.145	20.127	22.706	0.086	0.384
Mean	6.438	3.254	1.336	0.638	0.661	0.629	0.710	0.079	0.384

*Na*: Number of observed alleles; *Ne*: Number of effective alleles; *I*: Shannon’s Information Index; *Ho*: Observed heterozygosity; *He*: Expected heterozygosity; *Nm*: Gene flow; *PIC*: Polymorphic information content; *PI*: probability of identity; *PIsibs*: probability of identity among siblings

The *PIC* values of the 32 pairs of primers ranged from 0.400 (P4.2) to 0.827 (D11), with an average of 0.710. There were 30 pairs of primers with *PIC* values > 0.5, indicating that the screened primers had high polymorphism. These primers can effectively reveal the genetic diversity of the tested *Zanthoxylum* accessions and are suitable for DNA fingerprinting.

### Genetic relationship and cluster analysis of *Zanthoxylum* based on SSR markers

Genetic similarity coefficients (*GS*) are commonly used to evaluate the extent of genetic similarity among individuals. In this study, the genetic similarity coefficient matrix of 80 *Zanthoxylum* accessions was obtained using NTSYS-pc 2.1 software (Supplementary Figure S1). The *GS* values ranged from 0.0947 to 0.9868, with an average of 0.3864, indicating noticeable variation in the genetic backgrounds of the test materials. Notably, the *GS* value between ‘JJYH’ and ‘ZHJ’ was the smallest (0.0947), indicating that these two plants had the highest genetic variation and the furthest genetic relationship. Conversely, the *GS* value between ‘LZHHJ’ and ‘BSJ’ was the largest (0.9868), indicating that these two plants had very close genetic relationships. Additionally, the frequency distribution of the 3160 *GS*s obtained from the two-by-two comparison of the test samples revealed that the majority of the *GS*s fell within the range of 0.1 to 0.5, accounting for 77.5% of the total (Supplementary Figure S2). Among them, the largest number of *Zanthoxylum* accessions had *GS* values ranging from 0.1 to 0.2, accounting for 26.17% of the total. Overall, these results indicate that the 80 *Zanthoxylum* accessions possess a diverse range of genetic characteristics and a broad genetic background.

**Fig. 1:**
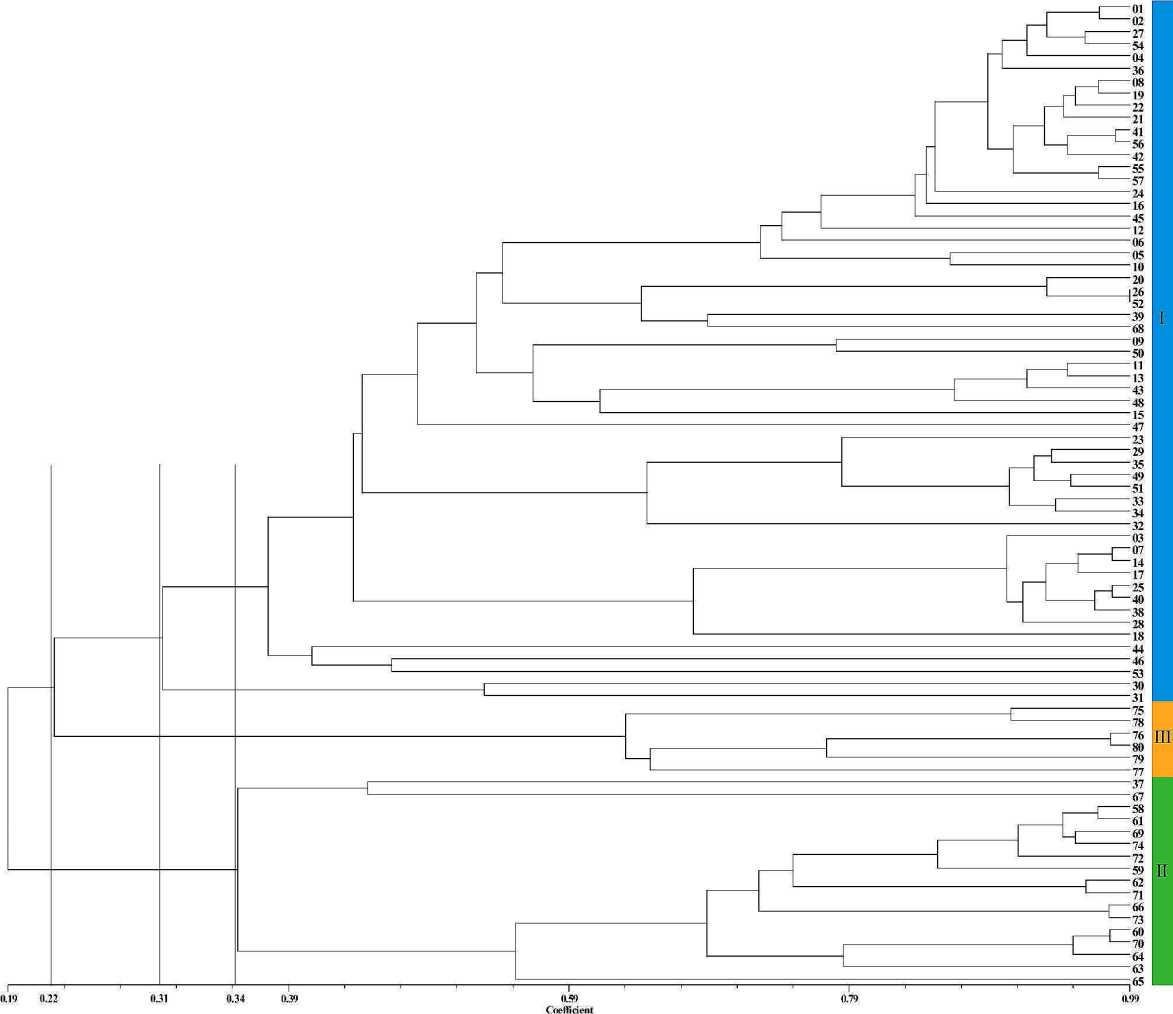
UPGMA clustering tree of 80 *Zanthoxylum* accessions based on SSR markers

The cluster analysis results demonstrated that using 32 SSR markers, it was possible to completely distinguish the 80 *Zanthoxylum* accessions (Fig. 1). With a *GS* threshold of 0.2217, the test accessions could be classified into three classes (I, II, and III). Class I consisted of 57 *Z. bungeanum* accessions, class II consisted of 17 *Z. armatum* accessions, and class III consisted of 6 *Z. piperitum* accessions. It is worth noting that “MYWCQHJ” (37) and “YJ” (67) in class II aggregate into a subclass at a *GS* value of 0.348. After calculation, it was found that the average *GS* values of “MYWCQHJ” and “YJ” with the other 15 *Z. armatum* accessions were 0.356 and 0.365, respectively, indicating that they have a distant genetic relationship with other *Z. armatum* accessions. Similarly, in class I, “HYWC ♂” (30) and " HYWC ♀” (31) clustered into a subclass at a *GS* of 0.312, showing a distant relationship with other *Z. bungeanum* accessions. Furthermore, we noticed that the GS values of “BSJ” (26) from Hebei and “LZHHJ” (52) from Henan amounted to 0.987, suggesting minimal genetic differences and a possible case of synonymy. Additionally, certain *Zanthoxylum* accessions from different source areas are clustered together, such as “LNDHP” (25) from Gansu and “MXLYJ” (40) from Sichuan, as well as “DJ” (27) from Gansu and “RCHJ” (54) from Shanxi. This clustering may be attributed to the frequent trade and introductions of *Zanthoxylum* between various regions. Moreover, the high correlation coefficient (0.977) calculated using the matrix comparison plot module of NTSYS-pc 2.1 software indicates the accuracy of the clustering results.

### Genetic diversity and differentiation of the *Zanthoxylum* population based on SSR markers

In this study, a total of 80 *Zanthoxylum* accessions were categorized into three populations based on the species: *Z. bungeanum* (Pop1), *Z. armatum* (Pop2), and *Z. piperitum* (Pop3). The genetic diversity analysis revealed that, among all three populations, Pop1 exhibited the highest *Na*, *Ne*, *Ha*, *He*, and *I* value (Table 3), suggesting that Pop1 possessed the highest genetic diversity. Pop2 had the second highest level, while Pop3 had the lowest. The coefficient of genetic differentiation (*Fst*) between the populations was calculated, yielding *Fst* values of 0.242 for Pop1 and Pop2, 0.335 for Pop1 and Pop3, and 0.429 for Pop2 and Pop3. The mean *Fst* was 0.335 (*Fst* > 0.25), indicating significant genetic differentiation between the three populations. AMOVA further demonstrated that genetic variation in *Zanthoxylum* species existed mainly within individuals (65%), with relatively little variation between populations (35%) (Table 4). Additionally, the average *Nm* was 0.629 (Table 2), suggesting limited gene exchange among individuals within each population, potentially attributed to the phenomenon of apomixis in *Zanthoxylum* species.

**Table 3:** The genetic diversity statistics among 3 populations of *Zanthoxylum* species

Pop	Na	Ne	I	Ho	He
Pop1	4.833	2.447	1.012	0.654	0.544
Pop2	3.694	1.982	0.769	0.556	0.415
Pop3	1.611	1.342	0.283	0.227	0.173
Total	10.139	5.771	2.064	1.437	1.132
Mean	3.380	1.924	0.688	0.479	0.377

*Na*: Number of observed alleles; *Ne*: Number of effective alleles; *I*: Shannon’s Information Index; *Ho*: Observation of heterozygosity; *He*: Expectation of heterozygosity

**Table 4:** The AMOVA of 3 populations of *Zanthoxylum* species

Source of variance	df	SS	MS	Variance component	Variation percentage %	*P* value
Among Pops	2	417.851	208.925	5.702	35%	< 0.001
Within Indiv	80	857.500	10.719	10.719	65%	< 0.001
Total	82	1275.351	-	16.421	100%	-

df: Degrees of freedom; SS: Sum of squares; MS: mean square

Furthermore, Nei’s genetic distance and genetic concordance study revealed that the genetic distance among the populations ranged from 0.854 to 1.190, with a mean value of 0.972. The genetic concordance ranged from 0.304 to 0.426, with a mean value of 0.383 (Table 5), indicating low genetic similarity and a high degree of genetic differentiation among the three populations. Pop2 and Pop3 exhibited the greatest genetic distance, representing the most distant relationship, whereas Pop1 and Pop2 displayed the smallest genetic distance, indicating a more recent relationship.

**Table 5:** Unbiased estimation of *Nei’s* genetic distance and genetic identity in 3 populations of *Zanthoxylum* species

Pop	Pop1	Pop2	Pop3
Pop1	-	0.854	0.872
Pop2	0.426	-	1.190
Pop3	0.418	0.304	-

Note: The upper right data represents Nei genetic distance, while the lower left data represents Nei genetic identity

**Fig. 2:**
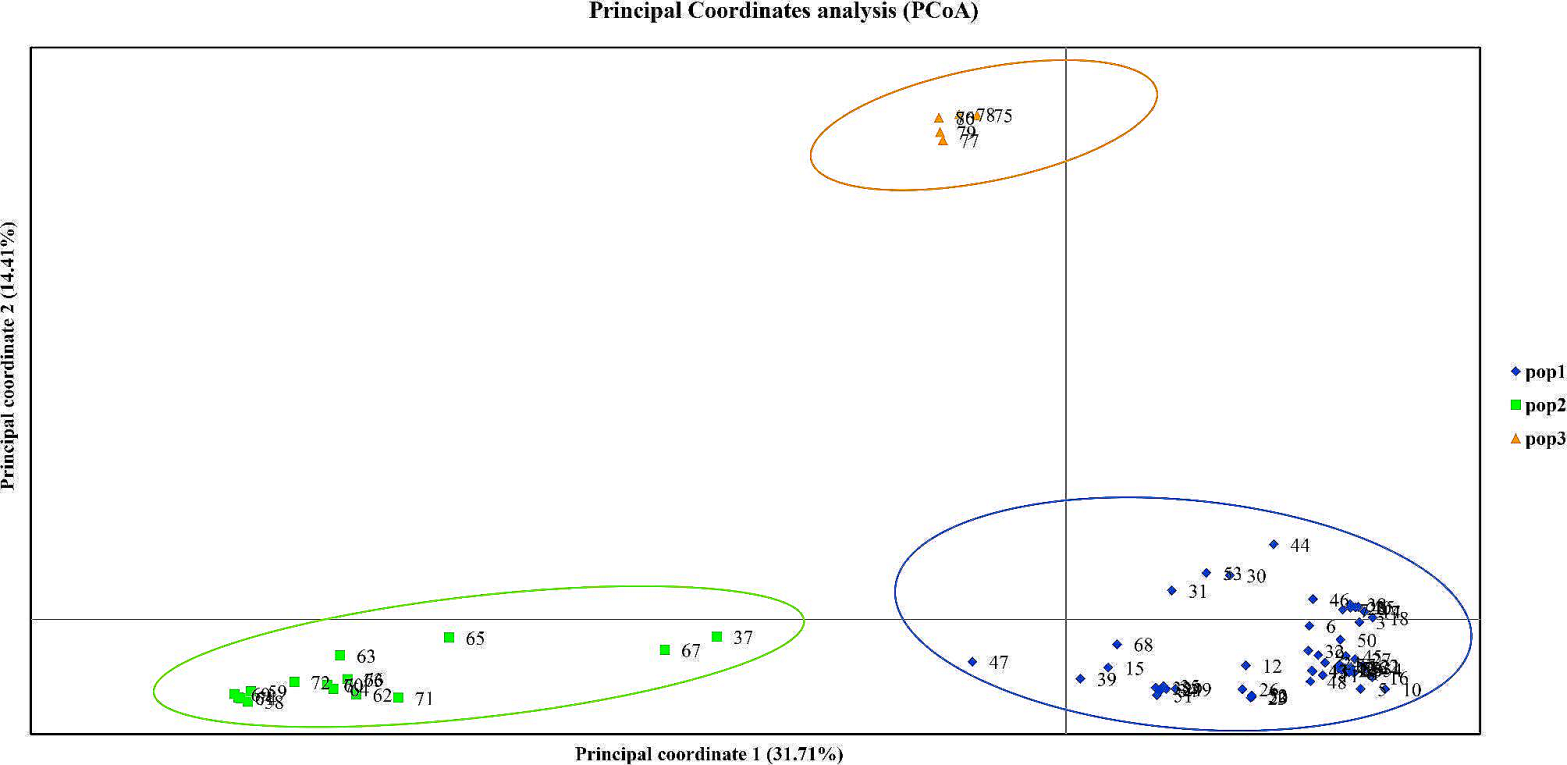
Principal coordinate analysis of 3 populations of *Zanthoxylum* species based on SSR markers

Principal coordinate analysis indicated that the first two principal coordinates accounted for 46.12% of the genetic variation among the 80 *Zanthoxylum* accessions. Principal coordinate 1 explained 31.71% of the variation, while principal coordinate 2 accounted for 14.41% (Fig. 2). The analysis classified the 80 *Zanthoxylum* accessions into three groups: the first group included 57 accessions of *Z. bungeanum*, the second group comprised 17 accessions of *Z. armatum*, and the third group consisted of 6 accessions of *Z. piperitum*. These findings were consistent with the results obtained from cluster analysis.

### Population structure analysis of *Zanthoxylum* based on SSR markers

**Fig. 3:**
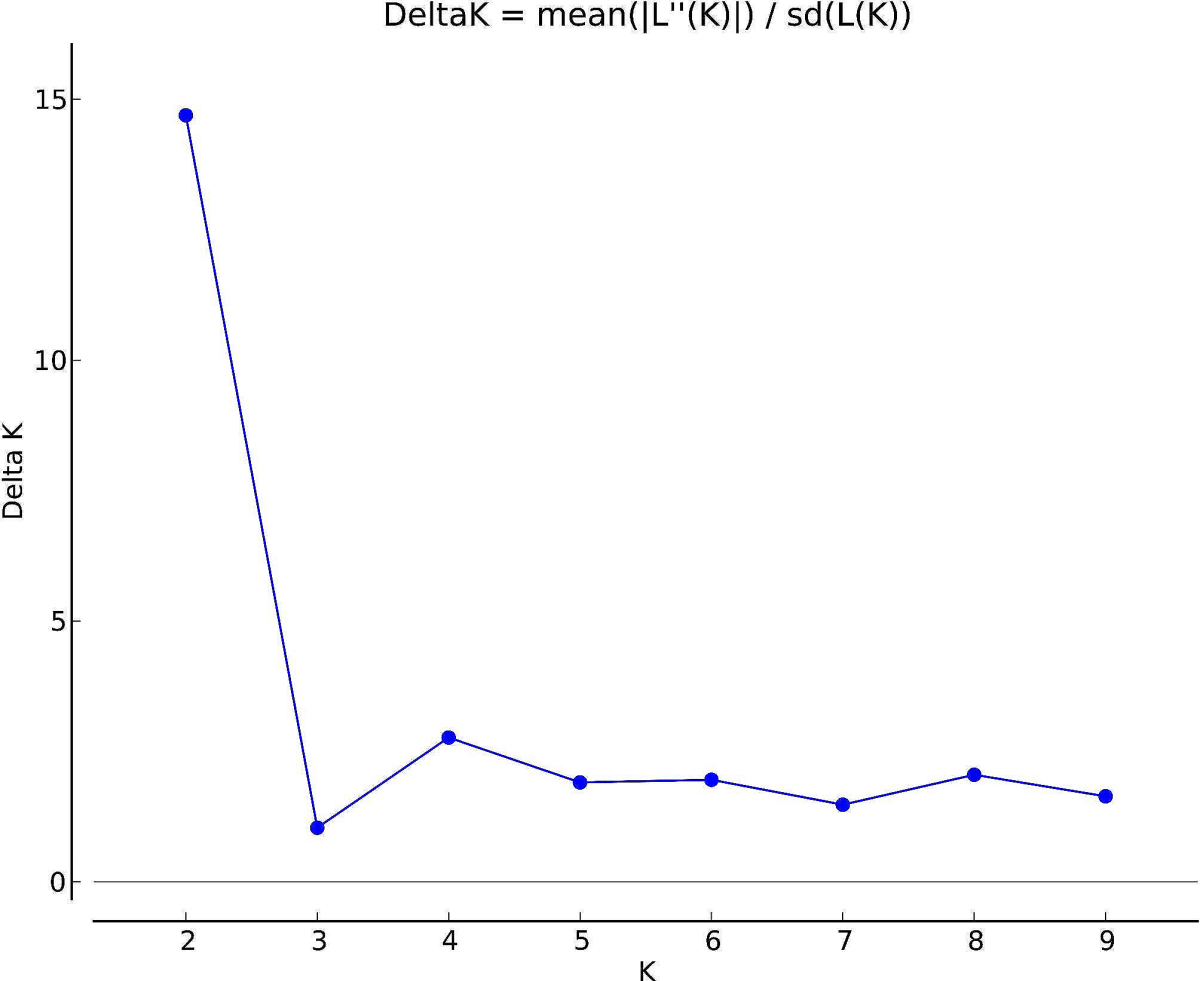
Delta K values for different numbers of populations assumed (K) in the STRUCURE analysis

**Fig. 4:**
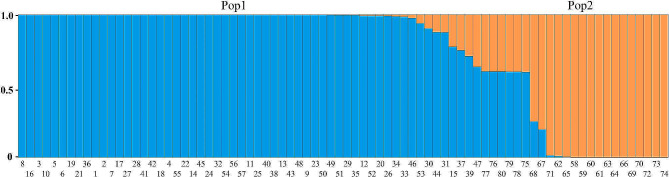
Population genetic structure of 80 *Zanthoxylum* accessions. Each rectangular column in the figure represents one accession, and the color and color scale of the columns represent the subpopulation to which it belongs and the proportion of the subpopulation it occupies (Blue represents Pop1, and Orange represents Pop2). The number on the X-axis is the accession number

In order to understand the genetic background and gene penetration of 80 *Zanthoxylum* accessions, the population structure of the test materials was analyzed by Structure software based on Bayesian modeling and the Q-values (Supplementary Table S2) (Pritchard et al., 2000) (probability that the i-th material has its genomic variation originating from the k-th subgroup) was counted. The results showed that Delta K has an optimal value when K = 2 (Fig. 3), therefore, the 80 *Zanthoxylum* accessions can be classified into 2 groups: Pop1 (blue) and Pop2 (orange) (Fig. 4); where Pop1 includes 63 accessions, mainly *Z. bungeanum* and *Z. piperitum*, and Pop2 includes 17 accessions, mainly *Z. armatum*.

Of the 80 *Zanthoxylum* accessions, 69 had Q-values ≥ 0.8, with a mean value of 0.99, indicating that these materials were from a single source, with a simple genetic background and a lack of genetic exchange between subgroups; 11 accessions had Q-values < 0.8 with a mean value of 0.66, suggesting that these materials possessed a mixed origin with a relatively complex genetic composition.

### Fingerprinting power of SSR markers and DNA fingerprint construction

*PI* is an important parameter for assessing the fingerprinting power of molecular markers, with lower values indicating higher fingerprinting efficiency of the markers [27]. According to the results in Table 2, the *PI* values of the 32 SSR markers ranged from 0.046 (D86) to 0.428 (P4.2), with an average value of 0.173. Assuming that all loci segregate independently, the probability of finding two random individuals with identical genotypes at the 32 marker loci is estimated to be 4.265 × 10^− 27^, i.e., it is almost impossible to find two different individuals with identical genotypes, suggesting that the markers developed in this study have strong fingerprinting power. *PIsibs* is considered to be the upper limit of *PI* [28], and the range of *PIsibs* values for the 32 SSR markers was 0.342 (D86) to 0.660 (P4.2), and the *PIsibs* value for all marker combinations was 1.282 × 10^− 11^.

**Fig. 5:**
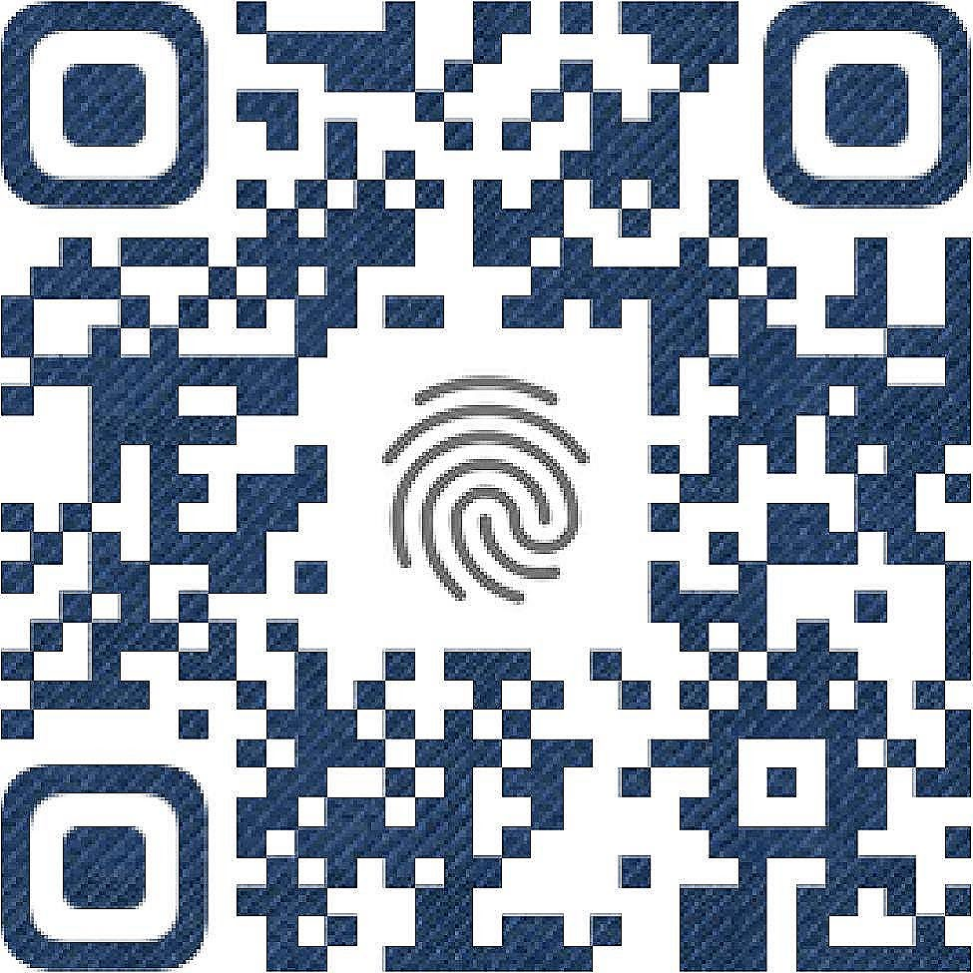
Fingerprint information of 80 *Zanthoxylum* cultivars based on SSR markers

Based on these results, combined with the results of primer amplification, eight SSR markers (D11, D23, D49, D81, D86, N63, P4.11, P4.17) with low *PI* values (the average value was 0.096) were screened to compose a core set of markers used to construct the fingerprinting of *Zanthoxylum*. Through the combination of these eight markers, 80 fingerprinting profiles with unique correspondences were obtained. The digital codes of 80 *Zanthoxylum* cultivars and their corresponding cultivar types, seed source locations and other information were merged to generate a QR code for fingerprinting (Fig. 5).

### iPBS primer screening and analysis of primer polymorphisms

Ten iPBS primers with high polymorphism and clear banding patterns were selected from a pool of 83 primers for analysis of genetic diversity in the 80 *Zanthoxylum* accessions (Supplementary Table S3).

A total of 127 bands were amplified from the ten selected primers, 120 of which were found to be polymorphic (Table 6). The number of bands per primer ranged from 4 to 21, with an average of 12.7 bands. The polymorphism ratio per primer ranged from 75 to 100%, with an average of 93.1%. The *PIC* values of the primers ranged from 0.201 to 0.324, with an average of 0.281. Notably, primer 2242 exhibited the highest level of polymorphism, with a *PIC* value of 0.324, while primer 2083 had the lowest level, with a *PIC* value of 0.201.

**Table 6:** The amplification results and genetic diversity index of 80 *Zanthoxylum* accessions by 10 iPBS primers

Primer	T	*N*	PPL (%)	PIC	Na	Ne	H	I
2083	7	7	100.0	0.201	2.0000	1.2456	0.1762	0.3030
2085	12	11	91.7	0.229	1.9167	1.2841	0.1928	0.3212
2222	16	13	81.3	0.294	1.8125	1.3923	0.2389	0.3707
2242	21	21	100.0	0.324	2.0000	1.4490	0.2704	0.4187
2243	16	16	100.0	0.312	2.0000	1.3966	0.2432	0.3804
2245	18	17	94.4	0.300	1.9444	1.4494	0.2737	0.4223
2271	14	14	100.0	0.282	2.0000	1.4129	0.2472	0.3861
2375	4	3	75.0	0.319	1.7500	1.4051	0.2390	0.3643
2380	9	8	88.9	0.287	1.8889	1.3538	0.2261	0.3579
2398	10	10	100.0	0.262	2.0000	1.3707	0.2381	0.3782
Total	127	120	-	2.811	19.3125	13.7595	2.3456	3.7028
Mean	12.7	12	93.1	0.281	1.9313	1.3760	0.2346	0.3703

*T*: Total number of amplified bands; *N*: Number of polymorphic bands; *PPL*: Polymorphism ratio; *PIC*: Polymorphic information content; *Na*: Number of observed alleles; *Ne*: Number of effective alleles; *H*: *Nei’s* genetic diversity; *I*: Shannon’s Information Index

### Genetic diversity analysis of *Zanthoxylum* based on iPBS markers

The genetic diversity indices of the 80 *Zanthoxylum* accessions were calculated with PopGene 1.32 software (Table 6), and the results showed that the mean values of Na, Ne, H and I were 1.9313, 1.3760, 0.2346 and 0.3703, respectively, indicating that the genetic variation among the 80 *Zanthoxylum* accessions was relatively high.

Genetic similarity coefficient matrices of 80 *Zanthoxylum* accessions were obtained via NTSYS-pc 2.1 software (Supplementary Figure S3). *GS* varied from 0.2206 to 1.0000, with an average of 0.5215; among them, the *GS* values of ‘MSQHJ’ and ‘HYWC ♂’, and ‘WCTJ’ and ‘HYWC ♂’ were all 0.2206, which indicated that they were the most distantly related. There were five groups of *Zanthoxylum* accessions with *GS* values of 1; these results, in combination with the SSR marker results, indicated that these materials were very close to each other and had highly similar genetic backgrounds; on the other hand, these results also indicated that the 10 iPBS markers in this study had limited discriminatory ability. Statistics on the frequency distribution of *GS* values of the test materials were found (Supplementary Figure S4), and the *GS* values were mainly distributed between 0.3 and 0.7, accounting for 74.56%, with the largest number of *Zanthoxylum* samples with *GS* values between 0.3 and 0.4 accounting for 27.09%.

### Cluster analysis of *Zanthoxylum* based on iPBS markers

**Fig. 6:**
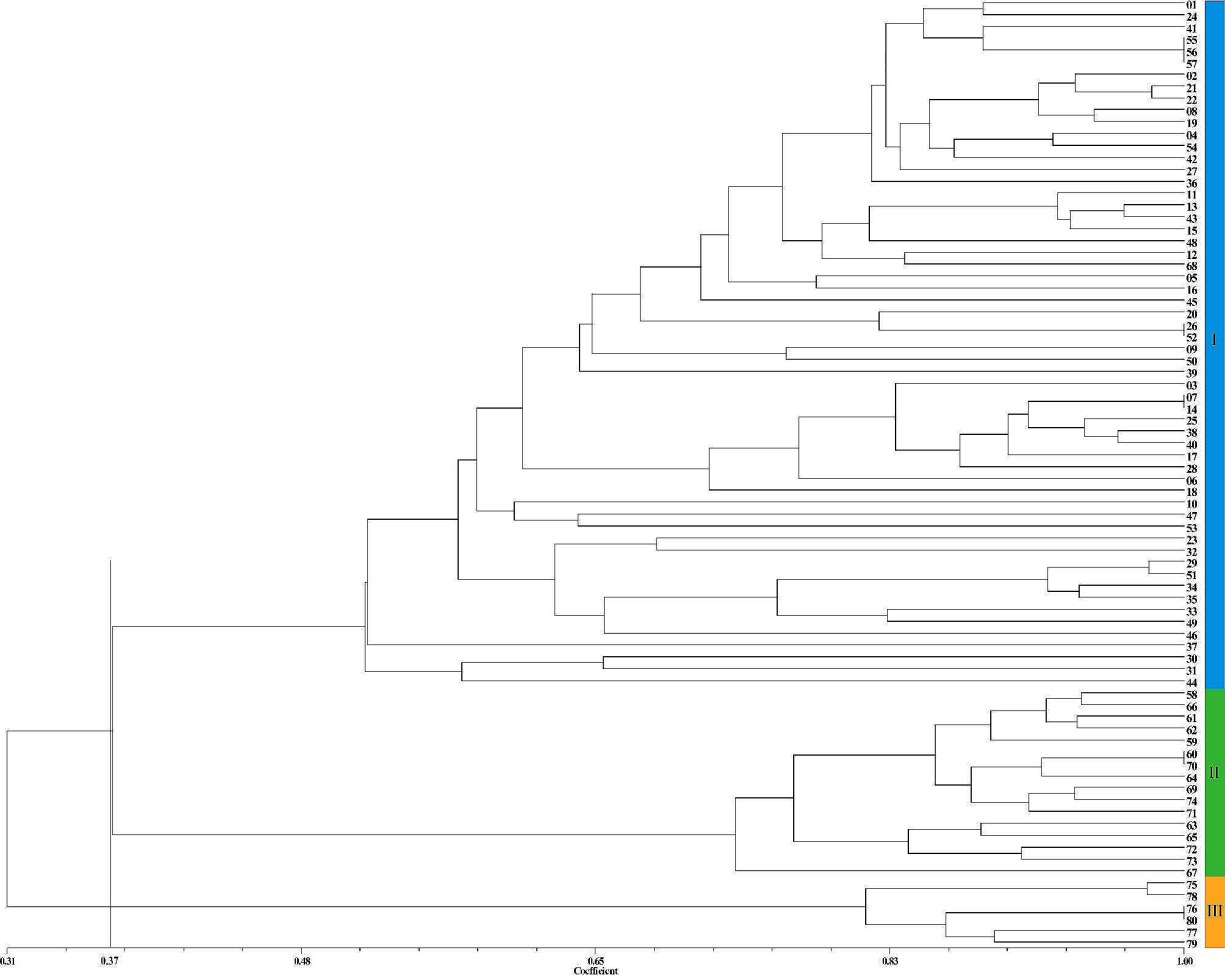
UPGMA clustering tree of 80 *Zanthoxylum* accessions based on iPBS markers

Based on the matrix of genetic similarity coefficients, a dendrogram depicting iPBS marker clustering of 80 *Zanthoxylum* accessions was constructed using the UPGMA method (Fig. 6). The analysis revealed that these 80 *Zanthoxylum* accessions could be categorized into three distinct groups, Group I, Group II, and Group III, representing *Z. bungeanum*, *Z. armatum*, and *Z. piperitum*, respectively, with a *GS* threshold of 0.3683. Notably, ‘MYWCQHJ’ did not cluster within any group associated with *Z. armatum*. This phenomenon may be attributed to two factors. First, this could be due to the limited number of iPBS markers utilized in this study. Second, this difference might be attributed to the unique characteristics of the ‘MYWCQHJ’ cultivar itself, as evidenced by its separate clustering within Group I. The correlation coefficient, computed using the Matrix comparison plot module in the NTSYS-pc 2.1 software, was found to be 0.966, underscoring the high accuracy of the clustering results.

**Fig. 7:**
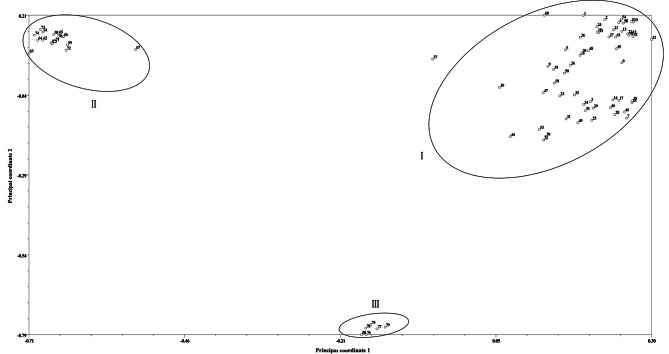
Principal coordinate analysis of 80 *Zanthoxylum* accessions based on iPBS markers

Furthermore, the principal coordinate analysis results concurred with the cluster analysis results. The 80 *Zanthoxylum* accessions were divided into three distinct categories (Fig. 7): the first category consisted of one accession of *Z. armatum* (‘MYWCQHJ’), 57 accessions of *Z. bungeanum*, the second category comprised 16 accessions of *Z. armatum*, and the third category included 6 accessions of *Z. piperitum*. This alignment between the two analyses strengthens the validity of the obtained classifications.

### Genetic and cluster analysis of *Zanthoxylum* based on SSR + iPBS markers

**Fig. 8:**
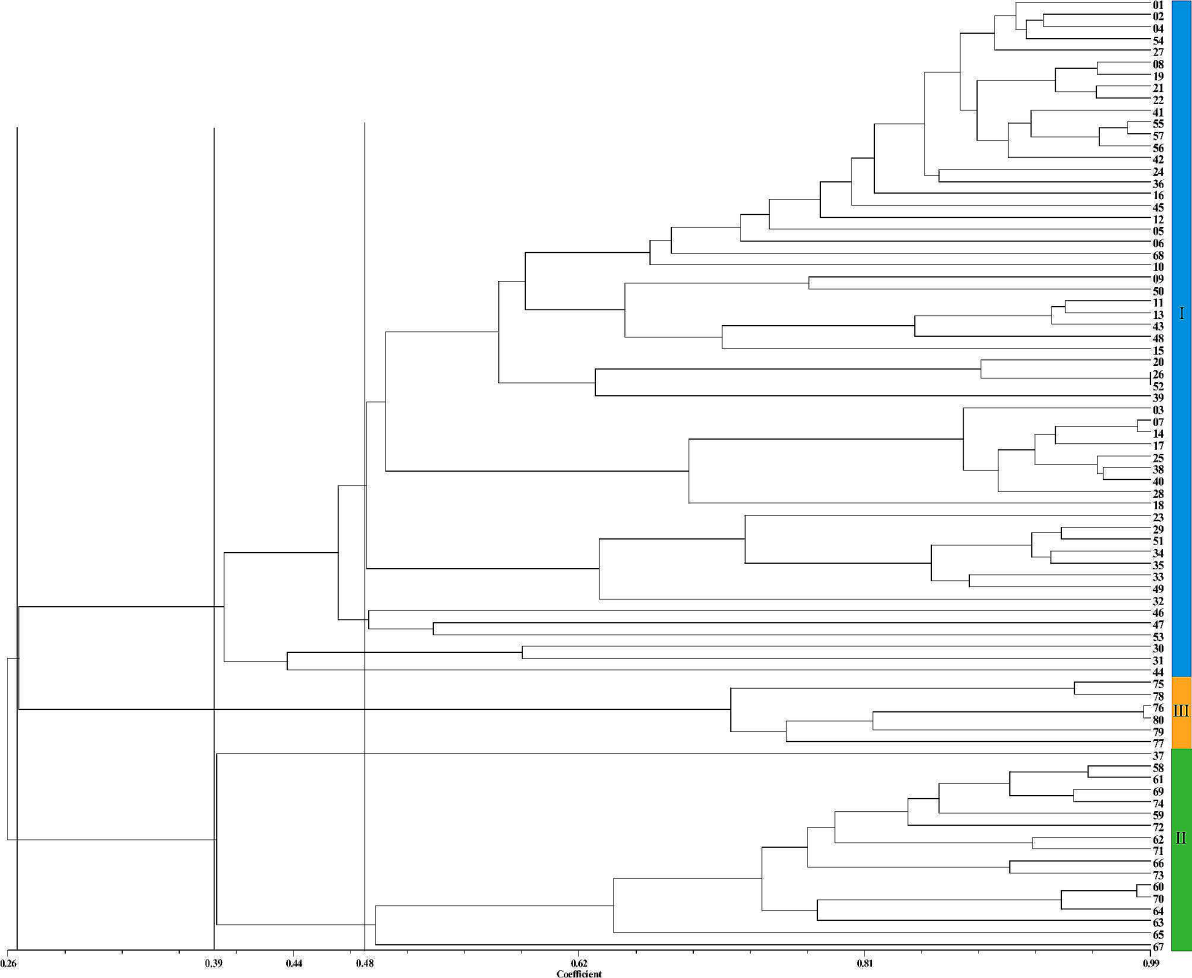
UPGMA clustering tree of 80 *Zanthoxylum* accessions based on SSR + iPBS markers

The genetic similarity coefficient matrix (Supplementary Figure S5) and clustering tree diagram (Fig. 8) were constructed through the integration of SSR and iPBS molecular marker data. The finding revealed that among the 80 *Zanthoxylum* accessions, the *GS* ranged from 0.1747 to 0.9921, with an average value of 0.4422. This indicates a significant disparity in the genetic backgrounds of the accessions. It should be noted that ‘HYWC ♂’ and ‘MSQHJ’ exhibited the lowest *GS* values (0.1747), while ‘BSJ’ and ‘LZHHJ’ demonstrated the highest *GS* values (0.9921). Among the *Z. bungeanum* species, ‘HYWC ♂’ and ‘XZHJ’ had the smallest *GS* values (0.3072), while ‘BSJ’ and ‘LZHHJ’ had the highest *GS* values (0.9921). In the case of *Z. armatum*, ‘MYWCQHJ’ and ‘LQYH’ had the smallest *GS* values (0.3611), while ‘MSQHJ’ and ‘WCTJ’ had the largest *GS* values (0.9833). Finally, within the *Z. piperitum* category, ‘JWCYH’ and ‘HSJ’ had the lowest *GS* values (0.6837), while ‘ZCSJ’ and ‘ZCSJ ♂’ had the highest *GS* values (0.9878).

Upon reaching a *GS* of 0.2657, the 80 *Zanthoxylum* accessions were divided into three classes: Class I represented *Z. bungeanum*, Class II represented *Z. armatum*, and Class III represented *Z. piperitum*. At a *GS* of 0.4856, Class I could be further divided into five subclasses. The first subclass comprised 43 *Z. bungeanum* cultivars, including all the accessions from Shaanxi (12/12), nearly all the accessions from Gansu (13/14), and almost half of the accessions from Sichuan (8/17). These three provinces are geographically close to each other and are major areas for *Zanthoxylum* production. The mixing of *Zanthoxylum* cultivars from these regions could be attributed to frequent introductions and resource exchange. Additionally, the first subclass included three cultivars from southwestern Yunnan and a few *Zanthoxylum* cultivars from northern regions, such as Hebei, Henan, Shandong, and Shanxi. The second subclass comprised eight *Zanthoxylum* cultivars, five from Sichuan, two from Hebei, and one from Gansu. The third subclass included ‘LWDHP’ and ‘LWXHP’ from Shandong and ‘PSDHP’ from Shanxi. The fourth subclass consisted of two special cultivars, ‘HYWC ♂’ and ‘HYWC ♀’, while the remaining ‘ZHJ’ accessions formed a separate fifth subclass. In Class II, ‘MYWCQHJ’ and ‘YJ’ were found to be distantly related to the other *Z. armatum* accessions and clustered into separate subclasses with *GS* values of 0.3909 and 0.4917, respectively. Overall, the clustering results revealed that the *Z. bungeanum* and *Z. armatum* cultivars from various source locations exhibited some mixing and were not exclusively clustered based on geographic differences. Clustering analysis utilizing only SSR or iPBS markers also confirmed this phenomenon. Conversely, combining the results of both markers provided a more accurate classification and effectively represented the genetic relationships among the tested *Zanthoxylum* accessions.

## Discussion

### Genetic diversity of *Zanthoxylum*

Genetic diversity serves as the foundation for the long-term survival and evolutionary advancement of species. The extent of genetic diversity within a species determines its evolutionary potential and ability to withstand adverse environmental factors [29]. In the case of plants, research on genetic diversity is crucial for comprehending the level of genetic variation and genetic structure within species. This serves as a significant indicator for evaluating the genetic potential of germplasm resources. Additionally, these findings could lead to resource utilization, germplasm innovation, and varietal improvement while also providing recommendations for resource conservation and management [30, 31].

Molecular markers represent an effective method for studying species genetic diversity. There are various types of molecular markers with different characteristics. By combining different molecular markers, researchers can examine different segments of the genome, thereby enhancing the coverage and uniformity of polymorphic loci. This approach compensates for any limitations and drawbacks associated with using a single type of molecular marker, enabling researchers to gain a comprehensive understanding of the species’ genetic information and enhancing the credibility of their findings [32].

The aim of this study was to assess the genetic diversity and relatedness among 80 *Zanthoxylum* accessions using SSR and iPBS molecular markers. SSR molecular markers are known for their superior variability and broad distribution within the genome. They are widely utilized across numerous genetic-related fields due to their codominance, high polymorphism, reproducibility, and consistent results [7]. In this study, we identified a total of 206 allelic variations among the 80 *Zanthoxylum* accessions using 32 selected SSR markers. Each marker displayed an average of 6.438 alleles (*Na*), an effective number of alleles (*Ne*) of 3.254, a Shannon’s information index (*I*) of 1.336, and *PIC* values ranging from 0.400 to 0.827, with an average of 0.710. Notably, 30 markers exhibited high polymorphism levels (*PIC* > 0.5). Among the genetic diversity indices, *Na* and the *PIC* are particularly important for assessing molecular marker polymorphisms [33]. In this study, the values for these two indices were greater than those reported by Li et al. [9] (*Na* = 3.5; *PIC* = 0.48) and Feng et al. [13] (*Na* = 4.636) in *Zanthoxylum*. Taken together, these findings indicate that the SSR markers employed in this study exhibited overall high polymorphism, revealing the genetic diversity of the tested *Zanthoxylum* accessions.

Compared to SSR molecular labeling technology, iPBS molecular labeling technology offers a simpler, faster, and more cost-effective approach. Throughout this study, 10 iPBS primers were employed to amplify a total of 127 bands across the 80 *Zanthoxylum* accessions. The average polymorphism rate of the primers was 93.1%. The *PIC* values ranged from 0.201 to 0.324, with an average of 0.281, indicating a moderate level of polymorphism, consistent with research findings in *Phoenix dactylifera* [34] (*PIC* = 0.287) and *Psidium guajava* [35] (*PIC* = 0.287). By combining the results of both sets of molecular markers, it was observed that the genetic diversity index obtained through iPBS markers was significantly lower than that obtained through SSR markers. This finding suggested that SSR markers possess greater polymorphism and are more suitable for analyzing the genetic diversity of *Zanthoxylum* germplasm resources. Such disparity is likely influenced by the number of markers used in this study; utilizing 32 SSR markers increases the likelihood of detecting greater genetic variation than does the use of only 10 iPBS markers. Moreover, SSR markers are codominant markers that distinguish between pure and heterozygous genotypes, thus conferring a greater advantage in revealing species genetic diversity than dominant markers. In summary, the utilization of both molecular markers revealed a considerable level of genetic diversity within the 80 *Zanthoxylum* accessions.

**Genetic relationship of*Zanthoxylum***.

The genetic similarity coefficient is a useful tool for evaluating genetic similarity. A higher genetic similarity coefficient indicates a closer genetic relationship and greater similarity between two individuals or groups, while a lower coefficient suggests greater genetic differentiation and greater genetic diversity [36]. Among the 80 *Zanthoxylum* accessions, the ranges of *GS* values obtained through the SSR, iPBS, and SSR + iPBS methods were 0.0947 ∼ 0.9868, 0.2206 ∼ 1.0000, and 0.1747 ∼ 0.9921, respectively, with statistically significant differences. The average *GS* values were 0.3864, 0.5215, and 0.4422, respectively, indicating relatively rich genetic diversity and a high level of genetic variation among the tested *Zanthoxylum* accessions. SSR markers exhibited a wider range of *GS* variation and smaller average *GS* values than did the other markers, suggesting that SSR markers are more effective at detecting genetic variation. The genetic relationships revealed by the two marker types were consistent. For instance, in the iPBS results, *GS* values of 1 were obtained between ‘FXDHP’ and ‘GJDHP’, ‘BSJ’ and ‘LZHHJ’, and ‘MSQHJ’ and ‘WCTJ’. These same groups also had relatively large *GS* values (0.9744, 0.9868, and 0.9730) according to the SSR results, indicating very close genetic relationships. This may be attributed to inconsistent naming of the same cultivar in different regions, known as the phenomenon of synonymy. In summary, both SSR and iPBS markers can be employed to assess the phylogenetic relationships of the *Zanthoxylum* species. However, SSR markers showed greater diversity and a more comprehensive reflection of the phylogenetic relationships, suggesting it has greater polymorphism. Additionally, SSR + iPBS markers compensated for the limitations of iPBS markers and provided a more accurate representation of the genetic relationships among the tested *Zanthoxylum* accessions. The cluster analysis findings also supported these conclusions. Based on the SSR, iPBS, and SSR + iPBS markers, the 80 *Zanthoxylum* accessions were divided into three categories (*Z. bungeanum*, *Z. armatum*, and *Z. piperitum*), and closely related *Zanthoxylum* species were grouped together. However, when iPBS markers were used, ‘MYWCQHJ’, which belongs to *Z. armatum*, was clustered with *Z. bungeanum* cultivars, indicating that SSR markers provided more accurate results. Furthermore, it is possible that the unique characteristics of ‘MYWCQHJ’ contributed to this clustering result, as evidenced by the presence of multiple unique loci or band patterns (Supplementary Figure S6). The calculated mean *GS* value of ‘MYWCQHJ’ compared to those of the other 16 accessions of *Z. armatum* was only 0.391 (based on SSR + iPBS markers), indicating a distant relationship. These findings highlight the unique genetic variation of ‘MYWCQHJ’, which may prove valuable in future efforts related to germplasm innovation and the development of new cultivars. Additionally, on the clustering tree diagrams of both markers, it was observed that some *Zanthoxylum* accessions from the same region were not clustered together (Fig. 8). These findings suggest that long-term cultivation, domestication of *Zanthoxylum* species, and trading and introduction between different regions may have contributed to this phenomenon. Notably, the single *Zanthoxylum* accession from Germany was not grouped separately but instead clustered together with Chinese *Zanthoxylum*, indicating a shared origin, consistent with previous research conducted by Feng [37].

### Genetic differentiation and genetic structure of *Zanthoxylum*

Gene differentiation (*Fst*) and gene flow (*Nm*) are crucial parameters for assessing genetic variation among populations, and they exhibit an inverse correlation wherein higher differentiation coefficients indicate lower levels of gene flow [38]. For *Fst*, the following categories are generally utilized: *Fst* ranges between 0 and 0.05, which suggests negligible genetic differentiation between populations; 0.05 and 0.15, which signifies a moderate degree of genetic differentiation; 0.15 and 0.25, which indicates a substantial degree of genetic differentiation; and *Fst* > 0.25, which signifies a high degree of genetic differentiation [39]. For *Nm*, it is generally accepted that *Nm* > 1 indicates that there is frequent gene exchange between populations, which prevents genetic differentiation of populations due to genetic drift and contributes to the maintenance of genetic stability of populations, while *Nm* < 1 indicates that gene flow is not sufficient to counteract the effects of genetic drift, thus contributing to the increase of genetic differentiation between populations [40]. In this study, we used SSR markers to analyze the genetic differentiation characteristics of three *Zanthoxylum* populations (Pop1, Pop2, and Pop3). The *Fst* values were 0.242, 0.335, and 0.429 between Pop1 and Pop2, Pop1 and Pop3, and Pop2 and Pop3, respectively, suggesting a high level of genetic differentiation among the three populations. Moreover, the mean *Nm* was 0.629 (< 1), indicating limited gene exchange among the populations. This can be attributed to the fusionless reproductive characteristics of *Zanthoxylum* species and the high levels of genetic differentiation among populations, which hinder gene flow [37]. Additionally, the AMOVA results indicated a high level of genetic differentiation among the tested *Zanthoxylum* accessions, with genetic variation predominantly arising within individuals (65%), while 35% of the genetic variation originated from between populations. Both cluster analysis and PCoA accurately categorized the 80 *Zanthoxylum* accessions into three groups corresponding to the three different *Zanthoxylum* species populations (Pop1, Pop2, and Pop3). The genetic analysis revealed substantial genetic distance (0.972) and low genetic concordance (0.383) among these three populations, further highlighting their high level of genetic differentiation. Geographical isolation is an important factor leading to population differentiation, due to environmental heterogeneity, genetic variation, and limited gene flow, resulting in the independent evolution of populations in different geographical regions [13, 41]. The distinct growth environments of these three groups contributed significantly to their differentiation, with *Z. armatum* found in frost-free regions of southwestern China characterized by warm and humid climates; *Z. bungeanum* exhibiting resilience and adaptability to wide areas with harsh climates (subtropical and temperate zones); mainly distributed in northern regions of the Qinling Mountains-Huaihe River in China [19]; and *Z. piperitum* concentrated in certain parts of Japan. Over an extended period, the combination of natural and artificial selection has limited genetic exchange between these *Zanthoxylum* populations, leading to significant differentiation. Generally, higher genetic diversity indicates greater complexity of plant diversity and greater potential for environmental adaptation [42]. Among the three populations, the *Z. bungeanum* population (Pop1) exhibited the highest genetic diversity, while the *Z. piperitum* population (Pop3) displayed the lowest. This discrepancy may be attributed to the number of samples and actual cultivars, as well as the stronger environmental adaptability and wider geographic distribution of *Z. bungeanum*. Consequently, *Z. bungeanum* germplasm resources can serve as crucial genetic breeding material for future cultivar selection and breeding endeavors.

Unlike the results of UPGMA cluster analysis and PCoA, Bayesian model-based population structure analysis classified the 80 *Zanthoxylum* accessions into two subgroups (Fig. 4), of which six *Z. piperitum* materials were not classified into a separate category. The reasons for this discrepancy have to do with the fact that the different methods take different computational approaches or provide different amounts of information [37]; on the other hand, it may be related to the small number of *Z. piperitum* material used in this study. Most of the 80 *Zanthoxylum* accessions (86%) had a single genetic component (Q-value ≥ 0.8), and only a few materials (14%) showed a mixture of both gene pools (Q-value < 0.8), suggesting a lack of genetic exchange between *Zanthoxylum* subgroups, which is consistent with the results of the analysis of population genetic differentiation.

### Construction of DNA fingerprint map and fingerprinting power

DNA fingerprinting is a molecular-level method used to identify different biological individuals by utilizing molecular markers. It is not influenced by environmental factors or by the developmental stage of organisms. In the case of plants, DNA fingerprinting is valuable for accurately and rapidly identifying cultivars, offering convenience for germplasm resource management, evaluation, protection of cultivar rights, and crop breeding [43]. Among several molecular markers, SSR markers are widely regarded as the preferred method for constructing plant DNA fingerprints. They have been recognized as one of the most powerful marker systems for identifying plant cultivar and have been successfully applied across multiple species [8, 43]. For instance, He et al. [44] established the genetic fingerprints of 33 standard flue-cured tobacco varieties using 48 SSR markers and developed identification technology for new tobacco varieties based on SSR markers. Chen et al. [43] created a DNA fingerprinting database of 128 excellent oil camellia cultivar using highly variable SSR markers.

*PI* and *PIsibs* are widely used as indicators of the fingerprinting power of molecular markers in studies of fingerprinting construction [28, 45]. In this study, the combined *PI* value of 32 SSR markers was 4.265 × 10^− 27^, and the low *PI* value showed high fingerprinting power. However, Waits et al. [28] argued that the assumption of independent segregation among sites does not hold because the substructure of plant populations is shaped by environmental and anthropogenic selection, leading to a possible overestimation of the theoretical *PI*, and thus *PIsibs* are usually used as a conservative upper limit for the *PI*; specifically, *PI* values of 1 × 10^− 4^ ∼ 1 × 10^− 2^ are considered sufficient for application to the identification of individuals in natural populations. The *PI* and *PIsibs* values in this study were much lower than the putative values, indicating that the 32 SSR markers have a very high potential for fingerprinting. Therefore, we combined eight pairs of primers to construct DNA fingerprints for 80 *Zanthoxylum* cultivars, each of which was assigned a unique numerical code. However, it should be noted that the number of *Zanthoxylum* cultivars that can be identified by this fingerprint method is limited. As the number of *Zanthoxylum* accessions used for identification increases and new cultivars are introduced and promoted, the number of new variant sites will increase as well. In such cases, timely and periodic updates to the fingerprint will be required to ensure its ongoing role in future research and application.

In comparison to SSR markers, iPBS markers have been less frequently employed to construct DNA fingerprints. Zeng et al. [46] successfully constructed fingerprints of 85 *Cymbidium goeringii* germplasm resources using two iPBS primers. Demirel et al. [47] used 17 iPBS markers to fingerprint and genetically analyze 151 potato genotypes. These studies demonstrated the feasibility of constructing plant fingerprints using iPBS markers. For our study, we selected 10 iPBS primers with high polymorphism and clear amplification bands from a pool of 83 primers. However, we found that these 10 iPBS markers were not sufficient to completely differentiate the 80 *Zanthoxylum* cultivars.

Notably, specific bands were observed in the amplification results for SSR markers, indicating that allelic loci, such as ‘HYWC ♂’, ‘HYWC ♀’, ‘MYWCQHJ’, and ‘YJ’, can serve as important molecular traits for cultivar identification (Supplementary Figure S6). Considering factors such as the ease of banding, number of available markers, polymorphic information content of the primers, and amplification stability, we believe that SSR markers are more suitable for constructing DNA fingerprints of *Zanthoxylum* species. However, it is important to acknowledge that iPBS markers have valuable potential when genomic information is lacking for a species. Moreover, for materials that are difficult to identify using a single molecular marker, a combination of multiple markers can improve identification efficiency.

Currently, with the decreasing cost of high-throughput sequencing technology, the construction of DNA fingerprints using SSR and/or SNP markers has become the most popular choice [48]. Future research can focus on the development of these two marker types, as well as the collection of more comprehensive *Zanthoxylum* germplasm resources, to construct a more perfect fingerprint map. This endeavor holds significant importance for the conservation and development of *Zanthoxylum* germplasm resources.

## Conclusions

This study aimed to assess the genetic diversity, genetic relationships, population genetic differentiation, and genetic structure of 80 *Zanthoxylum* accessions using 32 G-SSR markers and 10 iPBS markers. Additionally, a DNA fingerprint of *Zanthoxylum* cultivars was constructed. The findings of this research demonstrated that the 80 *Zanthoxylum* accessions exhibit a significant level of genetic diversity. Both the SSR and iPBS markers were effective at revealing the genetic relationship of *Zanthoxylum* species, with SSR markers providing a more comprehensive reflection of the genetic variation within the tested accessions. Moreover, limited genetic exchange was observed among the three populations of *Zanthoxylum*, resulting in noticeable genetic differentiation. In terms of discriminatory ability, SSR markers demonstrated greater strength than iPBS markers. Furthermore, the construction of DNA fingerprints for the 80 *Zanthoxylum* cultivars was achieved using eight pairs of SSR primers. These findings have significant implications for the conservation and utilization of *Zanthoxylum* resources, offering a valuable scientific foundation.

## Electronic supplementary material

Below is the link to the electronic supplementary material.

Supplementary Figure S1: Genetic similarity coefficient matrix heatmap of 80 *Zanthoxylum* accessions based on SSR markers

Supplementary Figure S2: Frequency distribution of genetic similarity coefficients of 80 *Zanthoxylum* accessions based on SSR markers

Supplementary Figure S3: Genetic similarity coefficient matrix heatmap of 80 *Zanthoxylum* accessions based on iPBS markers

Supplementary Figure S4: Frequency distribution of genetic similarity coefficients of 80 *Zanthoxylum* accessions based on iPBS markers

Supplementary Figure S5: Genetic similarity coefficient matrix heatmap of 80 *Zanthoxylum* accessions based on SSR + iPBS markers

Supplementary Figure S6: The amplification results of 80 *Zanthoxylum* accessions by SSR primers “D50” and “N76”

Supplementary Table S1: The sequence information of 32 pairs of SSR primers

Supplementary Table S2: Q-values of 80 *Zanthoxylum* accessions in two groups

Supplementary Table S3: The sequence information of 10 iPBS primers

## Data Availability

The datasets used and/or analysed during the current study are available from the corresponding author on reasonable request.
