# Supplementary figures and images for: Genetic diversity analysis and DNA fingerprint construction of Zanthoxylum species based on SSR and iPBS markers
Source: BMC Plant Biol. 2024 Sep 7;24:843. doi: 10.1186/s12870-024-05373-1 (PMC11380355; doi:10.1186/s12870-024-05373-1)

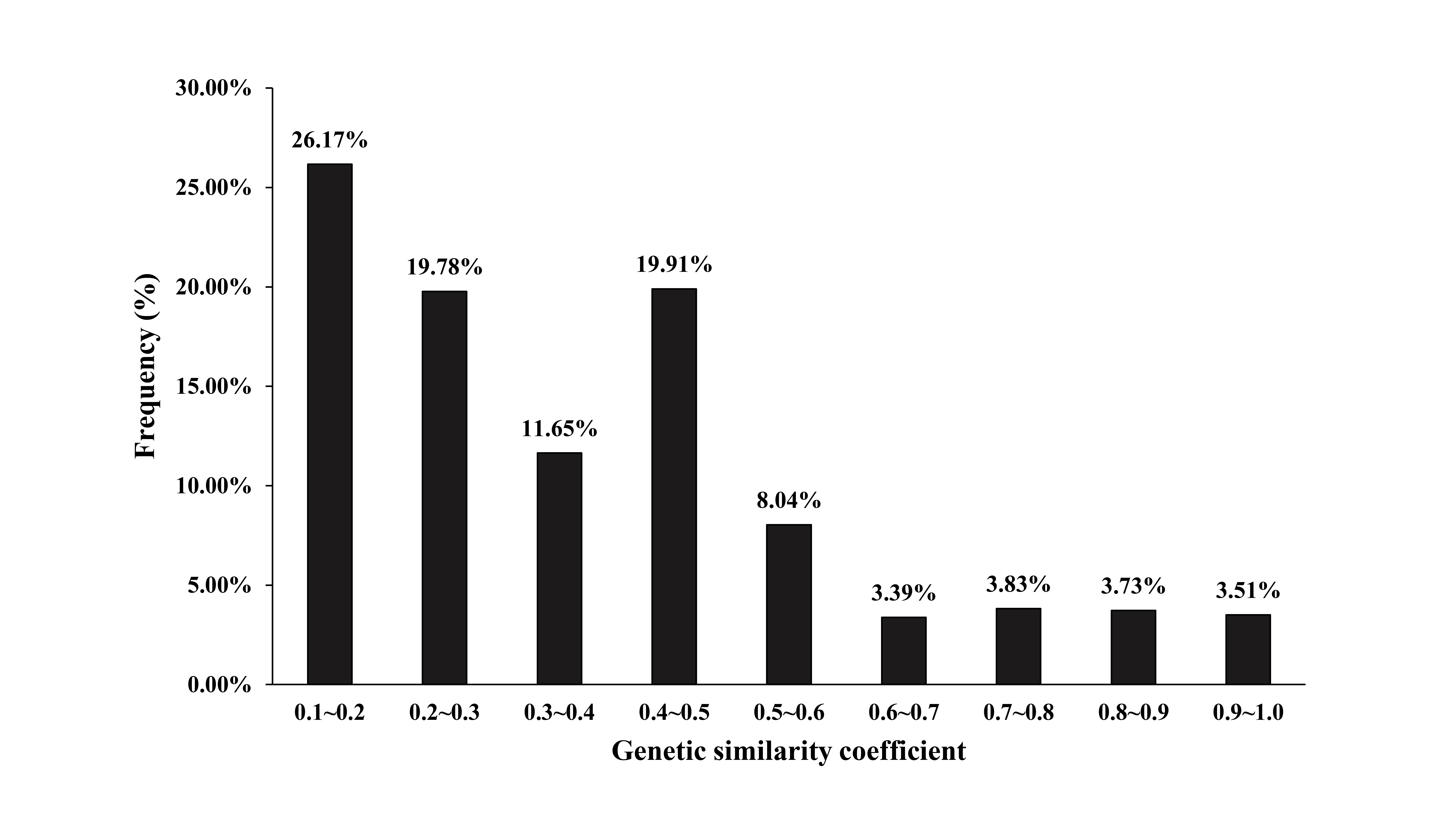

Supplement: Supplementary file 2 — Supplementary Figure S2: Frequency distribution of genetic similarity coefficients of 80 Zanthoxylum accessions based on SSR markers [file 12870_2024_5373_MOESM2_ESM.jpg]

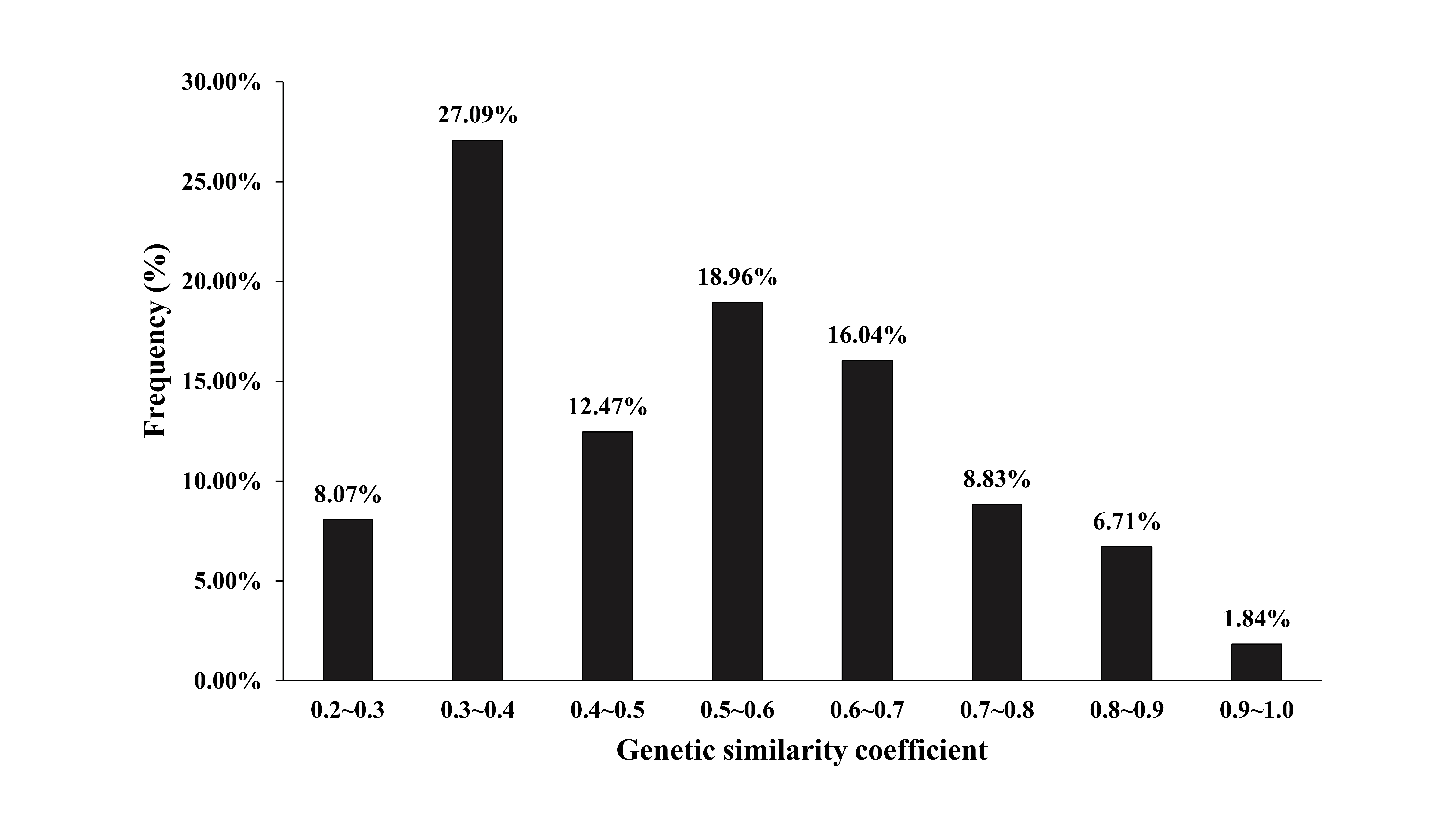

Supplement: Supplementary file 4 — Supplementary Figure S4: Frequency distribution of genetic similarity coefficients of 80 Zanthoxylum accessions based on iPBS markers [file 12870_2024_5373_MOESM4_ESM.jpg]

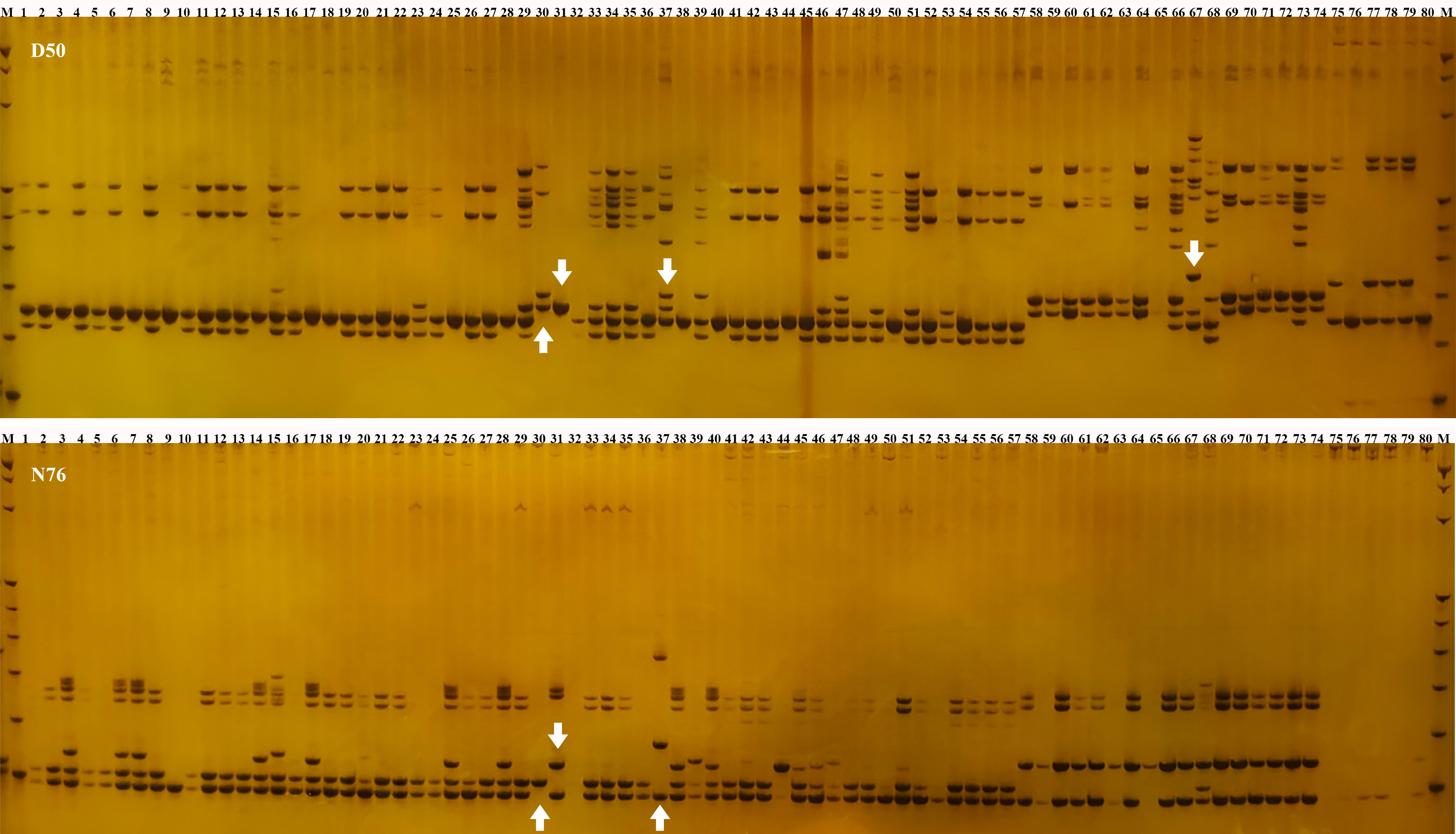

Supplement: Supplementary file 6 — Supplementary Figure S6: The amplification results of 80 Zanthoxylum accessions by SSR primers “D50” and “N76” [file 12870_2024_5373_MOESM6_ESM.jpg]
